# *Chlamydia trachomatis* Load in Population-Based Screening and STI-Clinics: Implications for Screening Policy

**DOI:** 10.1371/journal.pone.0121433

**Published:** 2015-03-31

**Authors:** Jeanne A. M. C. Dirks, Petra F. G. Wolffs, Nicole H. T. M. Dukers-Muijrers, Antoinette A. T. P. Brink, Arjen G. C. L. Speksnijder, Christian J. P. A. Hoebe

**Affiliations:** 1 Department of Medical Microbiology, Maastricht University Medical Center, School of Public Health and Primary Care, Maastricht, The Netherlands; 2 Department of Sexual Health, Infectious Diseases and Environmental Health, Public Health Service South Limburg, Geleen, The Netherlands; 3 Medical Microbiology Laboratory, Public Health Service Amsterdam, Amsterdam, The Netherlands; University of California Merced, UNITED STATES

## Abstract

**Objectives:**

If the *Chlamydia trachomatis* (CT) bacterial load is higher in high-risk populations than in the general population, this negatively affects the efficacy of CT screening incentives. In the largest retrospective study to date, we investigated the CT load in specimens collected from 2 cohorts: (1) attendants of a sexually transmitted infection (STI)-clinic and (2) participants of the Dutch population-based screening (PBS).

**Methods:**

CT load was determined using quantitative PCR in CT-positive male urine and female cervicovaginal swabs. CT loads were converted into tertiles. Using multinominal logistic regression, independent association of cohort, symptoms, risk behaviour and human cell count on load were assessed.

**Results:**

CT loads were determined in 889 CT-positives from PBS (n = 529; 71.8% female) and STI-clinics (n = 360; 61.7% female). In men, STI-clinic-cohort, human cell count and urethral discharge were positively associated with CT load. In women, PBS-cohort and cell count were positively associated with CT load. Both cohorts had the same range in CT load.

**Conclusions:**

The general population has a similar range of bacterial CT load as a high-risk population, but a different distribution for cohort and gender, highlighting the relevance of population-based CT-screening. When CT loads are similar, possibly the chances of transmission and sequelae are too.

## Introduction

*Chlamydia trachomatis* (CT) is the most common bacterial sexually transmitted infection (STI) worldwide [1, 2]. In the US alone, it has been estimated that there are 2–5 million new CT infections each year [3, 4]. The annual treatment costs for this disease and its complications, which include pelvic inflammatory disease, ectopic pregnancy and infertility is approximately $4 billion in the US, making these the most costly outcomes of any bacterial STI [4]. In the Netherlands, the number of cases of urogenital CT has been estimated at 80.000 infections in 2011, of which only a minority is diagnosed and treated [5–7]. In recent years, a steady increase in CT detection rates has been seen in the Netherlands, as in the rest of Europe. This can be attributed to increased diagnostic sensitivity and testing frequency [1, 2, 8], however, some studies suggest that this is also due to inadequate partner notification, changes in risk behavior of the individuals presenting for testing over time or even a loss of immunity after widespread early treatment [8–11]. The lack of symptoms in the majority of patients, the low perception of being at risk and the hurdle to get tested hampers timely detection and treatment of CT [12–14]. To overcome this, population register based screening for CT can be actualised, such as the Chlamydia Screening Implementation (CSI)-study in the Netherlands. CT screening programmes have two objectives; to reduce the chance of complications via early diagnosis and treatment, and to reduce the population prevalence and incidence [15]. Upon retesting, CT-positive samples from the CSI-trial were negative more frequently than expected, which typically indicates a load near the lower detection limit [Unpublished results. C. Hoebe, personal communication]. These results suggest that the CT load in CT-positives from population-based screening (PBS) might be lower than those diagnosed at clinical sites like STI-clinics. The effect of CT load on different clinical aspects, like transmission and the chance of sequelae is still under debate [16]. In trachoma and murine models, however, it has been demonstrated that a high CT load increases the transmission potential [17, 18], and the chance of sequelae [17–19]. Thus, the public health benefit per chlamydia-positive individual identified through screening is likely to be higher in those with higher loads because they are potentially at greater risk of developing sequelae and transmitting CT. Thus far, only a small study by Wiggins *et al*. [20] showed lower CT loads in community-based participants than those of symptomatic patients seeking treatment. Consequently, the purpose of the large and unique study presented here was to assess the CT load in low-risk CT-positive patients originating from the Dutch PBS and compare them to high-risk CT-positive patients visiting the STI-clinic. A second goal was to investigate a relationship between CT load and clinical symptoms.

## Materials and Methods

### Patient population and sample collection

CT-positive specimens from participants of the Dutch PBS-trial were included. This trial has been described in detail elsewhere [21]. In brief, specimens were collected at home in 2008–2011 from inhabitants of three distinct regions of the Netherlands. All men and women, between 16–29 years were asked to participate, while in one region (South Limburg) a risk-assessment was implemented first. Male respondents were asked to provide a first-void urine (FVU) specimen, and female respondents a self-collected vaginal swab or FVU. Within the three PBS-labs, CT diagnosis followed one of three different PCR-assays (BD ProbeTec, Beckton Dickinson, Franklin Lakes, USA; Gen-probe Aptima CT, Gen-Probe Inc., San Diego, USA; and COBAS Taqman, Roche Diagnostics, Basel, Switzerland) as per manufacturer’s protocol. CT-positive specimens were tested twice to confirm the test-result.

All CT-positive patients from the STI-clinic in South Limburg tested between October 2010—May 2012 were included. Samples were tested for CT with either COBAS Amplicor (Roche Diagnostics) or COBAS 4800 (Roche Diagnostics) as per manufacturer’s protocol.

Retrospective review of questionnaires and clinical data provided information about age, gender, ethnicity, sexual risk behaviour (number of sexual partners in the last 6 months and previous CT infection) and symptoms (present at least two days at time of sampling or in the previous month). Genitourinary symptoms for men were defined as urethral discharge or symptoms indicating a urinary tract infection (UTI; dysuria, urinary frequency and haematuria), and for women symptoms included vaginal discharge, intermenstrual bleeding, postcoital bleeding, abdominal pain and UTI-symptoms.

Every patient was included only once, if they were 16–30 years of age, and completed the questionnaire. Because the most predominant samples were FVU for males and cervicovaginal swabs for females, other sample-types were excluded. Patients were excluded if they reported HIV-positivity or antibiotic use in the previous month. All *Neisseria gonorrhoea* and *Mycoplasma genitalium* positive samples were excluded from the study to prevent possible bias in assessing the relationship between load and symptoms.

### Ethics statement

After reviewing the protocol within the context of the Medical Research Involving Human Subjects Act, the local Medical Ethics Committee of the Maastricht University Medical Center (METC azM/UM) approved this study and waived the need for informed consent (METC 13-4-026).

### Nucleic Acid extraction

Total nucleic acids from 200μl sample were isolated using the QIAamp DNA Mini kit (Qiagen, Hilden, Germany), and eluted in 120 μl. The eluate was stored at -20°C and thawed once for quantification. Prior to DNA-isolation an internal extraction and amplification control was added to all samples and to each a negative extraction control, as described elsewhere [22, 23].

### CT qPCR

Quantitative PCR for CT used primers targeting the single-copy *OmpA* gene, coding for the major outer membrane protein, as described by Jalal *et al*. [24]. For eukaryotic cell determination, primers targeting the MHC class II antigen (*HLA-DQA1*) gene were used, as described by van der Helm, *et al*. [25], allowing normalization of the CT load and a test for adequacy of the sample.

For absolute quantification, the *ompA*- and *HLA-DQA1* PCR product were cloned separately into the pGEM-T easy vector (Promega Corporation, Madison, WI, USA) according to the manufacturer’s protocol. Plasmids were isolated using alkaline lysis and purified using phenol/chloroform extraction as described previously [26]. Dilutions covering a range of 5 log were made (corresponding to 10^6^ to 10^2^ copies/ml in clinical samples). QPCR was performed with a 7900HT Real-Time PCR System (Applied Biosystems, Foster City, California). Each run, the 96-micro-wells plate contained both dilution-series, a negative control and the samples for quantification.

PCR amplification was performed in a total volume of 25 μl, consisting of 10 μl DNA and 15 μl reaction mixture containing 12.5 μl Absolute qPCR Rox Mastermix (Thermo Scientific, Waltham, USA) and 2.5 μl primer/probe mix consisting of 840 nM forward and reverse primer and 100 nM probe. The amplification reaction consisted of 15 minutes of initial activation at 95°C, followed by 42 cycles of 95°C for 15 seconds and 60°C for 60 seconds.

### Load determination

Cycle threshold-values were entered into the master curve (calculated from over 10 dilution series), and then exponentially transformed to achieve an absolute value (CT/ml). Samples were deemed inadequately sampled and excluded from further analysis when no human cells were detected or no target could be detected at all. Samples where only *HLA* was detected were included as they are critical to answering the hypothesis about low load samples. The initial diagnostic CT-screening was done with assays targeting the chlamydial plasmid or RNA, which are both present in relative abundance to the single-copy *OmpA*-gene used in our load assessment. Furthermore, the original CT-screening was done in duplicate for all PBS samples to diminish false positivity. We assigned these ‘low load’ samples, which were proven positive at the initial screening, a load at half the lowest detection limit. This is a generally accepted method to deal with values below the detection limit [27].

### Statistical analysis

Statistical analyses were stratified by gender due to different specimen types with distinct load distributions per specimen. Descriptive analyses included CT and human cell load range, age, ethnicity (Caucasian/non-Caucasian), sexual history, prior CT infection and clinical symptoms. Bacterial load was converted into three categories (low/middle/high) for analyses, as assumptions for normality were not met using load as a continuous variable. The lowest load category consisted of samples with a CT load below the quantification limit of our assay, while the middle and highest load category contained an equal number of samples in which the load could be detected. Multinomial logistic regression was performed to assess determinants of load, with the lowest category as reference category. First, unadjusted analyses were performed for bacterial load categories and each determinant separately. Subsequently, all determinants with p≤0.1 were included in the multinomial logistic regression model in a forward stepwise fashion to calculate adjusted odds ratios. All analyses were adjusted for age and ethnicity. As we were interested in patient origin this was included in the model regardless of the p-value in multivariable analysis. All multivariable analyses are shown with and without the inclusion of human cell load. Results were considered statistically significant at p≤0.05. All statistical tests were performed using the IBM SPSS Statistics for Windows, version 20.0 (IBM Corp. Armonk, NY, USA).

## Results

### Study population

Inclusion criteria were met by 889 patients. The PBS cohort comprised 529 patients, of which 149 (28.2%) were male. From the STI-clinic, 360 patients were included, of which 138 (38.3%) were male. Table 1 gives an overview of other patient characteristics, such as age, ethnicity, sexual risk behaviour and symptoms.

**Table 1:** Patient characteristics.

		**Men**		**Women**	
		***PBS***	***STI-clinic***	***PBS***	***STI-clinic***
		*N (%)*	*N (%)*	*N (%)*	*N (%)*
**Age (years)**	16–20	30 (20.1)	26 (18.8)	109 (28.7)	84 (37.8)
	21–25	73 (49.0)	89 (64.5)	179 (47.1)	118 (53.2)
	26–30	46 (30.9)	23 (16.7)	92 (24.2)	20 (9.0)
**Ethnicity**	Caucasian	77 (51.7)	114 (82.6)	241 (63.4)	206 (92.8)
	*missing*	2 (1.3)	0	0	3 (1.4)
**Sexual partners previous 6**	0–1	62 (41.6)	32 (23.2)	178 (46.8)	87 (39.2)
**months**	2–3	41 (27.5)	64 (46.4)	135 (35.5)	95 (42.8)
	>4	36 (24.2)	42 (30.4)	50 (13.2)	38 (17.1)
	*missing*	10 (6.7)	0 (0.0)	17 (4.5)	2 (0.9)
**Prior CT infection**	Yes	21 (14.1)	18 (13.0)	64 (16.8)	30 (13.5)
**Mean cells (log**_**10**_ **); [range]**		3.7	4.1	5.9	6.3
		[1.1–6.9]	[1.5–6.0]	[1.0–7.6]	[1.8–7.7]
**Symptoms** ^**a**^					
**Any**	Yes	22 (14.8)	51 (37.0)	183 (48.2)	74 (33.3)
**UTI-symptoms**	Yes	18 (12.1)	57 (35.5)	101 (26.6)	45 (20.3)
***Women***					
**Intermenstrual bleeding**	Yes			41 (10.8)	12 (5.4)
**Postcoital bleeding**	Yes			36 (9.5)	13 (5.9)
**Vaginal discharge**	Yes			103 (27.1)	40 (18.0)
**Abdominal pain**	Yes			53 (13.9)	29 (13.1)
***Men***					
**Urethral discharge**	Yes	4 (2.7)	18 (13.0)		

^a^ only symptoms associated with CT were included in the analyses.

Abbreviations: PBS, population-based screening; STI, sexually transmitted infection; CT, *Chlamydia trachomatis*, UTI, urinary tract infection.

### Bacterial load

Five samples (0.4%) were excluded due to inhibition of the PCR reaction, and 70 samples (5.8%) were excluded due to a lack of human cells in the sample, as shown by a negative *HLA-DQA1* PCR, making adequate sampling unlikely and load normalisation impossible. CT quantification within 42 cycles was possible for 697 samples (78.4%). CT load ranged from 15–8.5x10^7^ and 15–4.9x10^4^ CT/ml in female swabs and male FVU respectively.

### CT load in correlation with covariates in men

On univariable analysis, STI-clinic men had a significantly increased odds of being in the middle or high load category compared to men from the PBS cohort (Table 2), who were most often in the low load category. The amount of cells was significantly associated with the CT load; every increase in the log_10_ number of cells increased the odds of the CT bacterial load being in the middle or highest load category. When experiencing any symptom, urethral discharge or UTI-symptoms, men were significantly more often in the high load category than in the low load category. Indirect measures of sexual behaviour, such as the number of sexual partners in the last 6 months or a prior CT infection, were not associated with the bacterial load. On forward stepwise multivariable analysis, the cohort, number of cells and urethral discharge were independently associated with the bacterial load. When HLA was not included in multivariable analysis, the cohort and the presence of symptoms were independently associated with the CT load.

**Table 2:** Determinants of load-classification in men.

		**Univariable**						**Multivariable** ^1^				**Multivariable** ^2^			
		**Middle** ^a^			**High** ^a^			**Middle** ^a^		**High** ^a^		**Middle** ^a^		**High** ^a^	
		OR ^b^	95% CI	OR ^b^		95% CI	aOR ^b^ ^c^	95% CI	aOR ^b^ ^c^		95% CI	aOR ^b^ ^d^	95% CI	aOR ^b^ ^d^	95% CI
**Patient Origin**	PBS	1		1			**1**		1			**1**		1	
	STI-clinic	2.78*	1.48–5.14	2.02*		1.09–3.73	**2.34** *	**1.20–4.54**	1.38		0.68–2.78	**2.80** *	**1.47–5.31**	1.68	0.89–3.20
**Sexual partners**	0–1	1		1											
**previous 6 months**	2–3	1.26	0.64–2.49	1.27		0.67–2.40									
	≥4	1.47	0.75–2.91	1.56		0.74–3.27									
**Prior CT infection**	No	1		1											
	Yes	1.32	0.58–3.01	1.06		0.46–2.48									
**Nr. of cells (log**_10_ **)**		2.08^#^	1.54–2.81	3.29^#^		2.32–4.65	**2.01** ^#^	**1.47–2.73**	**3.34** ^#^		**2.34–4.77**				
**Symptoms**															
**Any**	No	1		1								1		**1**	
	Yes	1.24	0.62–4.46	2.32*		1.22–4.41						0.94	0.46–1.92	**2.01** *	**1.03–3.91**
**UTI-symptoms**	No	1		1											
	Yes	1.30	0.64–2.64	2.24*		1.16–4.35									
**Urethral discharge**	No	1		1			1		**1**						
	Yes	1.30	0.36–4.69	3.48*		1.17–10.35	1.19	0.31–4.57	**4.27** *		**1.23–14.88**				

N = 285 for both uni- and multivariable analysis. Multivariable significant results are shown in bold.

^1,2^ Two separate multivariable analyses were performed.

^a^ Reference category: low.

^b^ adjusted for age and ethnicity.

^c^ adjusted for nr. of cells, patient origin and urethral discharge.

^d^ adjusted for patient origin and the presence of symptoms.

* *p* ≤ 0.05.

# *p* ≤ 0.001.

### CT load in correlation with covariates in women

STI-clinic women had a lower odds of being in the middle load category compared to women from the PBS on univariable analysis and a similar odds of being in the high load category (Table 3). With every increase in the log_10_ number of cells, the chances of being in the highest load category simultaneously increased. Neither symptoms, nor indirect measures of sexual behaviour were associated with the bacterial load in women. On multivariable analysis, both the cohort and the amount of cells remained independently associated with the load. PBS women had a higher chance of being in the middle load category, yet an equal odds of being the highest load category as STI-clinic women, while an increasing amount of cells increased the odds of being in the highest load category. Excluding HLA from the multivariable analyses did not alter the results.

**Table 3:** Determinants of load-classification in women.

		**Univariable**				**Multivariable** ^1^				**Multivariable** ^2^						
		***Middle*** ^a^		***High*** ^a^		***Middle*** ^a^		***High*** ^a^		***Middle*** ^a^			***High*** ^a^			
		OR ^b^	95% CI	OR ^b^	95% CI	aOR ^b^ ^c^	95% CI	aOR ^b^ ^c^	95% CI	aOR ^b^ ^d^	95% CI	aOR ^b^ ^d^		95% CI	
**Patient Origin**	PBS	1		1		**1**		1		**1**			1			
	STI-clinic	0.43*	0.24–0.76	0.82	0.47–1.45	**0.43** *	**0.24–0.76**	0.67	0.37–1.19	**0.43** *	**0.24–0.76**		0.82		0.47–1.45	
**Sexual partners**	0–1	1		1												
**previous 6 months**	2–3	0.85	0.49–1.48	0.98	0.56–1.70											
	>4	1.05	0.47–2.35	1.21	0.55–2.69											
**Prior CT infection**	No	1		1												
	Yes	0.77	0.40–1.51	0.77	0.39–1.50											
**Nr. of cells (log**_10_ **)**		0.94	0.78–1.16	1.67^#^	1.29–2.15	1.00	0.80–1.23	**1.70** ^#^	**1.31–2.20**							
**Symptoms**																
**Any**	No	1		1												
	Yes	1.07	0.63–1.81	1.21	0.71–2.04											
**UTI-symptoms**	No	1		1												
	Yes	1.05	0.56–1.93	1.12	0.61–2.06											
**Intermenstrual**	No	1		1												
**bleeding**	Yes	0.98	0.38–2.55	1.38	0.55–3.48											
**Postcoital bleeding**	No	1		1												
	Yes	0.60	0.25–1.44	0.78	0.33–1.84											
**Vaginal discharge**	No	1		1												
	Yes	1.09	0.58–2.04	1.27	0.69–2.37											
**Abdominal pain**	No	1		1												
	Yes	1.17	0.54–2.56	1.16	0.53–2.53											

N = 599 for both uni- and multivariable analysis. Multivariable significant results are shown in bold.

^1,2^ Two separate multivariable analyses were performed.

^a^ Reference category: low.

^b^ adjusted for age and ethnicity.

^c^ adjusted for nr. of cells, patient origin and urethral discharge.

^d^ adjusted for patient origin

*p ≤ 0.05.

#*p* ≤ 0.001.

### Human cell load

**Fig 1:**
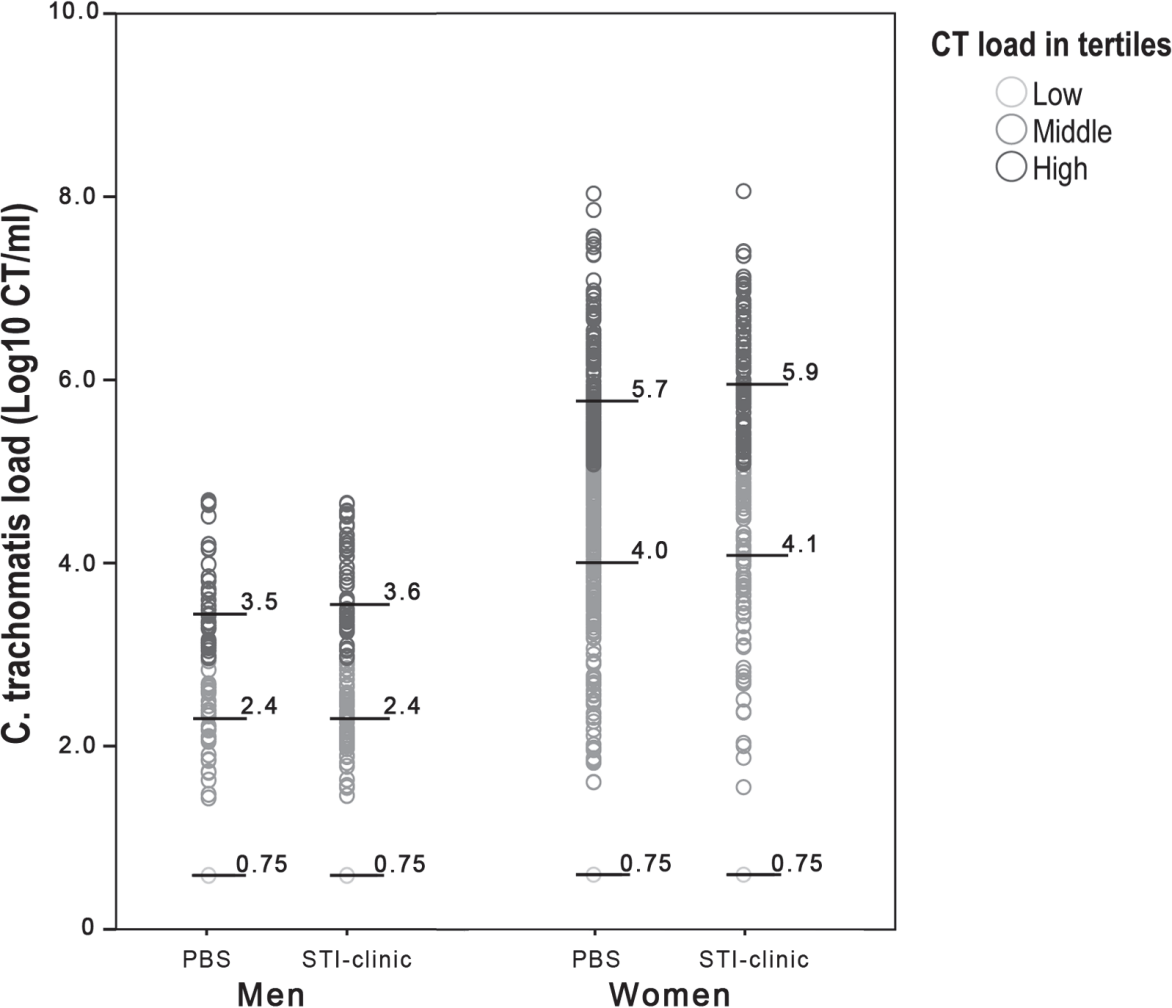
CT load distribution per load category for men and women. Low load samples make up the low load category. All other samples were divided equally between the middle and high load category. Black horizontal bars depict the median of each load category. Number of patients in each cohort; PBS men: low = 75, middle = 33, high = 41; STI-clinic men: low = 40, middle = 53, high = 45. PBS women: Low = 42, middle = 187, high = 151; STI-clinic women: low = 35, middle = 75, high = 112. Abbreviations: PBS, population-based screening; STI, sexually transmitted infection; CT, *Chlamydia trachomatis*.

Load distribution per patient group for the three groups of bacterial loads is shown in Fig 1. FVU contained maximally 7.0x10^6^ human cells and cervicovaginal swabs contained a maximum of 5.0x10^7^ human cells per ml.

Although we cannot differentiate between leukocytes and epithelial cells with our PCR-assay, we investigated if the number of human cells present was associated with symptoms. We found that the number of human cells was unrelated to the presence of symptoms (OR 1.19 [95% CI 0.9–1.5]) or urethral discharge in men (OR 1.03 [95% CI 0.7–1.5]), both in the STI-clinic and PBS population.

## Discussion

With nearly a thousand patients, this is the largest study to date to quantify CT load and it is the only study to investigate CT-positives from PBS (which provide a low-risk community) and clinical sites like STI-clinics (a high-risk population) simultaneously. This study was done to investigate the hypothesis that the bacterial load in CT-positives from PBS might be lower than those from STI-clinics. Low CT loads may decrease the transmission potential and the risk of negative outcomes of this disease, negatively affecting the efficacy of population based screening initiatives implemented in e.g. the UK and USA. In CT animal models [18, 19, 28], and in other STI’s like HCV [29], HIV [30, 31], HPV [32] it has already been demonstrated that high loads negatively impact outcome, and increase the chance of transmission. Our results show that STI-clinic women had a lower odds of being in the middle load category, yet similar odds of being in the high load category as PBS women. Contrastingly, PBS men had a bacterial load in the low load category more frequently than the men from the STI-clinic, whose load was more frequently in the middle category, and loads were similar in the high load category.

Taken together these results form a complex outcome, and not enough is known about the replication dynamics in humans to be able to predict how the CT load evolves over time in individuals. Therefore we propose two theories to explain the observed results.

In women, we propose that the observed differences in CT load in the different cohorts may be due to different sampling moments during the infection, as hypothesized by Price *et al*. [33]. Hypothetically, in the case that CT would follow similar replication dynamics observed in animal models [34], STI-clinic women are actively seeking testing soon after exposure to CT, while PBS women, apparently unaware of their exposure, get tested later in the CT replication curve [33].

In men, our results also point toward a correlation between the CT load and symptoms (consistent with other studies [35, 36]). The most likely explanation for the association of higher CT loads with symptoms may be that a higher bacterial load induces a greater inflammatory response. This is consistent with the observation that FVU from STI-clinic men contained more cells than the urine from PBS men (data not shown). Furthermore, *in vitro* data shows that CT intracellular infections induce pro-inflammatory cytokines and chemokines [37, 38]. Thus it is theoretically likely that a greater load would be associated with a greater inflammatory response [39]. It is surprising that some men in the community with high loads and thus probably a high inflammatory response do not seek care. However, it is possible that up to 40% of men with urethritis do not notice their urethral discharge [40, 41].

Similar bacterial CT loads in PBS- and STI CT-positives are pertinent to both male and female chlamydia screening efforts, as an equally high load in both populations likely makes the chance of transmission and complications equally high [17–19, 28]. These results should provide an incentive to increase efforts to engage more women in population-based CT screening programs. In men, the implications of these results are less evident, as the bacterial load in PBS men is more often low than that of STI-clinic men, but the CT load can reach identical heights. Thus, there are asymptomatic men with high bacterial loads present in the community. It is unknown how long they remain infectious for, and during this time they are likely to be at a high risk of transmitting CT [17–19]. Therefore it is important to include both asymptomatic and symptomatic men in screening programmes, both because of their own health risk, but also for the risk of transmission and subsequent sequelae in their sexual partners.

Most other studies investigating CT load excluded samples with a load below the quantification limit. Our study has the same percentage of samples with a bacterial load below the detection limit of the assay than other studies with the same or comparable PCR target [20, 35, 42]. The relatively high load reported in most other studies investigating load, can probably be explained through the exclusion of unquantifiable samples, and due to the inclusion of too few samples. As an example, Michel *et al*. [36], reported higher CT loads for men (mean log 4.1) in STI-clinic men than is found in our study (mean log 2.13). However, for women, the CT loads are in the same range (mean log_10_ 4.1 EB’s/ml) as our data (mean log_10_ 4.2 bacterial copies/ml). Wiggins *et al*.[20], the only other study to investigate a low-risk population, quantified geometric mean (gm) 1755 CT copies/ml in male FVU, to our gm of 136 copies/ml. In women, Wiggins *et al*.[20], found a gm of 10829 copies/ml (log_10_ 4.03) per female swab to our 18482 copies/ml (log_10_ 4.2). Gomes *et al*. [42], who also took the human cell load into account, found a higher bacterial load of <0.2–3.8 log_10_ (CT/100cells) in FVU, than the values of -2.5–4.6 log_10_ (CT/100cells) in this study. The wide variety of loads presented is reflected in the diversity of methods and samples used to investigate bacterial load [16, 20, 24, 25, 35, 36, 42–45]. More universally acknowledged protocols in CT load determination are needed.

A possible source of bias in this study is the use of CT-positives from the PBS. Although derived from the general population, they might not be entirely representative thereof. Participation in PBS is associated with many factors connected to CT-positivity, such as >2 partners in previous 6 months, no long-term relationship, a non-Dutch partner and STI symptoms [46]. Although pre-selection of high-risk patients took place in South Limburg, analyses with only patients from South Limburg (PBS versus STI-clinic) did not show different results. Thus, regardless of the selection that took place before participation, these CT-positives are drawn from the general population.

A limitation of our study is that we did not assess the CT genotype distribution. Nevertheless we think that the impact of this limitation on our results is negligible as previous studies [42, 47, 48] have not demonstrated a clear relationship between genotypes and the CT load. Furthermore, the inclusion of samples with a load too low for quantification, restricted analyses of our data as they made parametric testing impossible. Several alternatives have been evaluated before choosing the use of tertiles, such as Poisson regression and linear regression analyses, which all showed comparable results.

A general limitation encountered in CT load studies is the lack of knowledge about the duration and load curve of a CT infection. Both are likely influenced by the innate and protective immune response, which will vary between individuals. Here, we assumed that CT replication in humans follows the qualitatively similar replication dynamics observed in animal models [34], but this hypothesis warrants further investigation. Samples with a high bacterial load may be presumed to be sampled at the height of infection, while lower loads may indicate the beginning or resolution of a CT infection. It is also possible that some patients have a persistent chronic infection [49, 50], where a low load remains stable over a long period of time, and it is uncertain how this chronic infection influences the outcome of the disease, as infected cells produce inflammatory cytokines which might also result in complications like oviduct scarring [37]. With regard to transmission too, too little is known about the effect of the infectious dose to warrant any definite statements. The chance of CT transmission per sexual act is estimated to be 10% [51], but this is likely influenced by the CT load, as it is in mice [18, 19]. Thus the transmission per episode of sexual intercourse may vary between individuals but not necessarily between partnerships as they may have multiple episodes of sexual intercourse. This would be interesting to investigate in mathematical modelling studies. While we looked at behavioural factors influencing bacterial load, we expect that other (micro)biological factors, local microbiome and host factors, also affect CT load.

In short, we demonstrated that for CT-positive men STI-clinic-cohort, human cell count and urethral discharge were positively associated with CT load. In women, PBS-cohort and cell count were positively associated with CT load. The range of CT loads between cohorts was the same for both men and women, but the distribution within the cohorts differed. These results support the importance of CT screening efforts, based on CT load, to detect PBS CT-positives not yet diagnosed and treated, as no difference in the height of the bacterial load can be demonstrated. The clinical implications of CT load need further investigation.
